# Recent advances in Raman probes for multiplexed bioimaging

**DOI:** 10.1186/s41232-025-00400-6

**Published:** 2025-12-02

**Authors:** Takaya Togo, Hiroyoshi Fujioka, Mako Kamiya

**Affiliations:** Laboratory for Chemistry and Life Science, Institute of Integrated Research, Institute of Science Tokyo, Kanagawa, 226-8501 Japan

**Keywords:** Raman imaging, Label-free, Live-cell, Clinical applications, Multiplex detection, Functional probe

## Abstract

Bond-selective vibrational imaging techniques, such as Raman spectroscopy, are opening up many applications that were previously considered impossible or inaccessible by other means, such as fluorescence imaging. In particular, vibrational microscopy offers unique advantages, such as the ability to perform highly multiplexed, label-free imaging. Indeed, recent advances in optical and chemical technologies have made it possible to image biological phenomena at the cellular level with high sensitivity, high resolution, and high specificity. Applications of vibrational microscopy both in biological research and in medicine, including the detection of pathological lesions, are expanding rapidly. Here, we provide a general overview of Raman microscopy, and we review recent progress in cutting-edge applications, including label-free imaging and the development of small Raman tags, Raman probes enabling highly sensitive ultra-multiplexed observation, and functional Raman probes.

## Introduction

Fluorescence microscopy has been widely used by life scientists to image biological phenomena, but recently vibrational imaging methods such as Raman imaging have been attracting increasing attention due to their label-free nature [1]. In particular, the past decade has witnessed rapid progress in the development of vibrational probes with unique characteristics, including inherently small size, sharp spectral features, sensitivity to local environments, and multiplex imaging capability [2–4]. Vibrational spectroscopy encompasses infrared spectroscopy and Raman spectroscopy, but here we focus on Raman microscopy.

The Raman effect was first observed in 1928 by Sir C.V. Raman, for which he was awarded the Nobel Prize in Physics in 1930 [2]. However, due to the extremely low intensity of Raman signals, there were few applications of the effect until the 1970 s, when plasma ion gas lasers became available [3, 4]. It was anticipated that Raman probes would be smaller in size and provide sharper spectral characteristics compared to bulky fluorescent probes [5], but the technical limitations of microscopes and poor biocompatibility prevented widespread introduction at that time. However, the emergence of microscopes utilizing nonlinear optical effects (stimulated Raman scattering, SRS [6–11]; coherent anti-Stokes Raman scattering, CARS [12–14]) provided robust technologies with orders-of-magnitude higher sensitivity and speed. Since 2013 in particular, the combination of SRS with vibrational probes has been advanced by several groups [15–24]. Such innovations have enabled investigations of many biomolecules and metabolites [15–31], leading to a range of applications in the biomedical field [32–38].

In this review, we first provide an overview of the technique, describing how initial weaknesses have been addressed by the technical improvement of optical systems, and we then review the latest advances in label-free imaging, small Raman tags, Raman probes for ultra-multiplexed observation, and functional Raman probes.

## Development of Raman microscopy

**Fig. 1:**
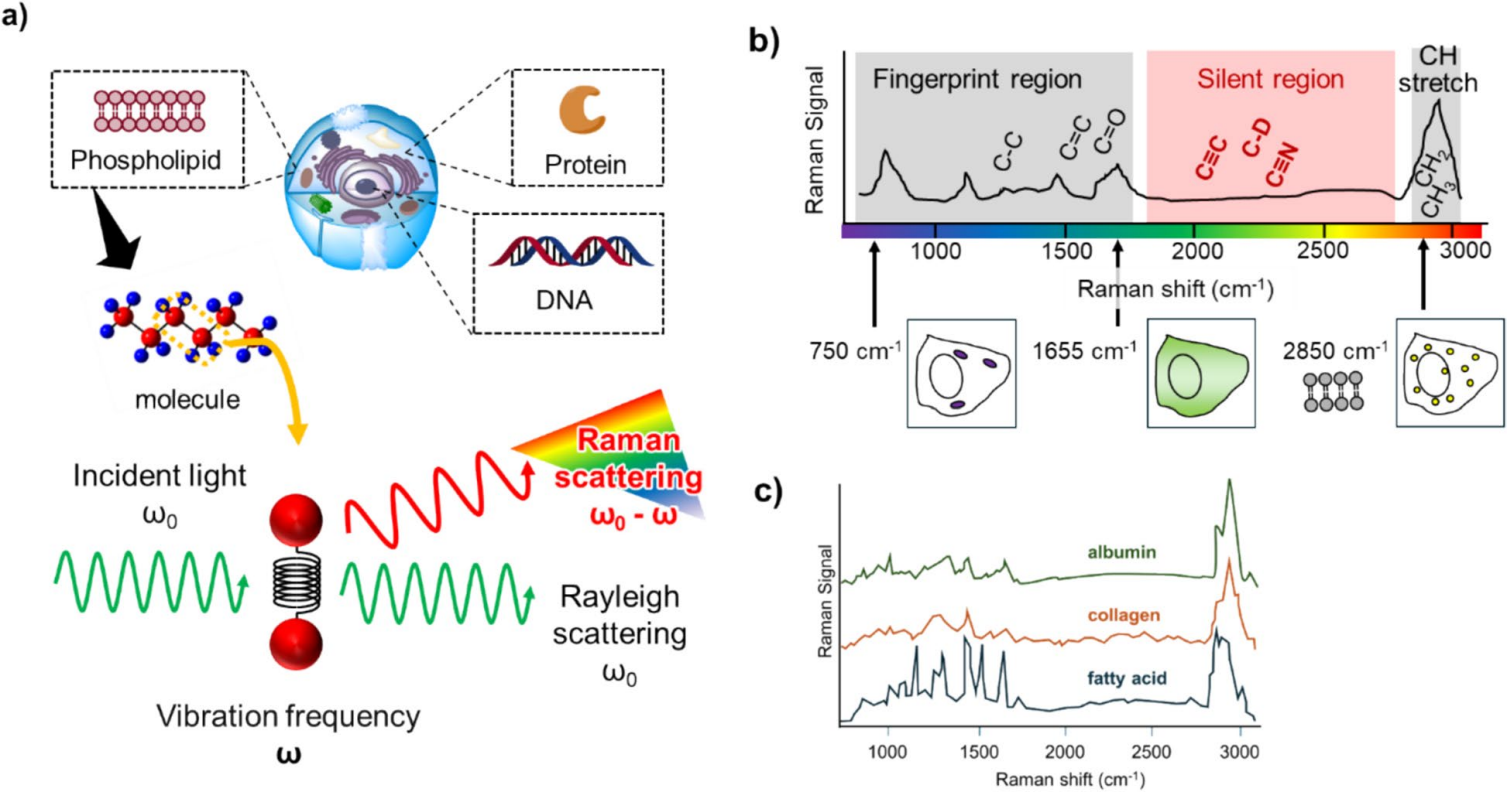
Overview of Raman imaging. **a** When the light is incident onto a material, most of this scattered light has the same vibrational frequency as the incident light (ω_0_, Rayleigh scattering), but some fractions of scattered light have different frequency to the incident light (ω_0_—ω, Raman scattering). The Raman shift is the difference in frequency between incident light and Raman scattering, which corresponds to the vibrational frequency (ω) of the chemical bond contained in the samples. **b** Example of Raman spectrum of cells, which can be roughly divided into three regions: fingerprint region, silent region, and CH stretching region. Functional groups present in biological samples appear in the fingerprint region and the CH stretching region, while signals arising from endogenous biological molecules are inherently absent in silent regions. Thus, functional groups such as alkynes which show characteristic vibrational peak in this region can be used tags for imaging with low background signal. By utilizing the characteristic Raman wavenumbers in fingerprint region and CH stretching region, some intracellular structures can be visualized. For example, mitochondria can be visualized by acquiring a Raman image at 750 cm^−1^, which originates from cytochrome c contained in mitochondria. The distribution of proteins can be visualized by acquiring a Raman image at 1655 cm^−1^, which is attributed to the amide-I vibration of proteins. The distribution of lipids can be visualized by acquiring a Raman image at 2850 cm^−1^, which is attributed to CH_2_ vibrations primarily contained in lipids. **c** Examples of Raman spectra of specific biomolecules (albumin, collagen, and fatty acid)

**Fig. 2:**
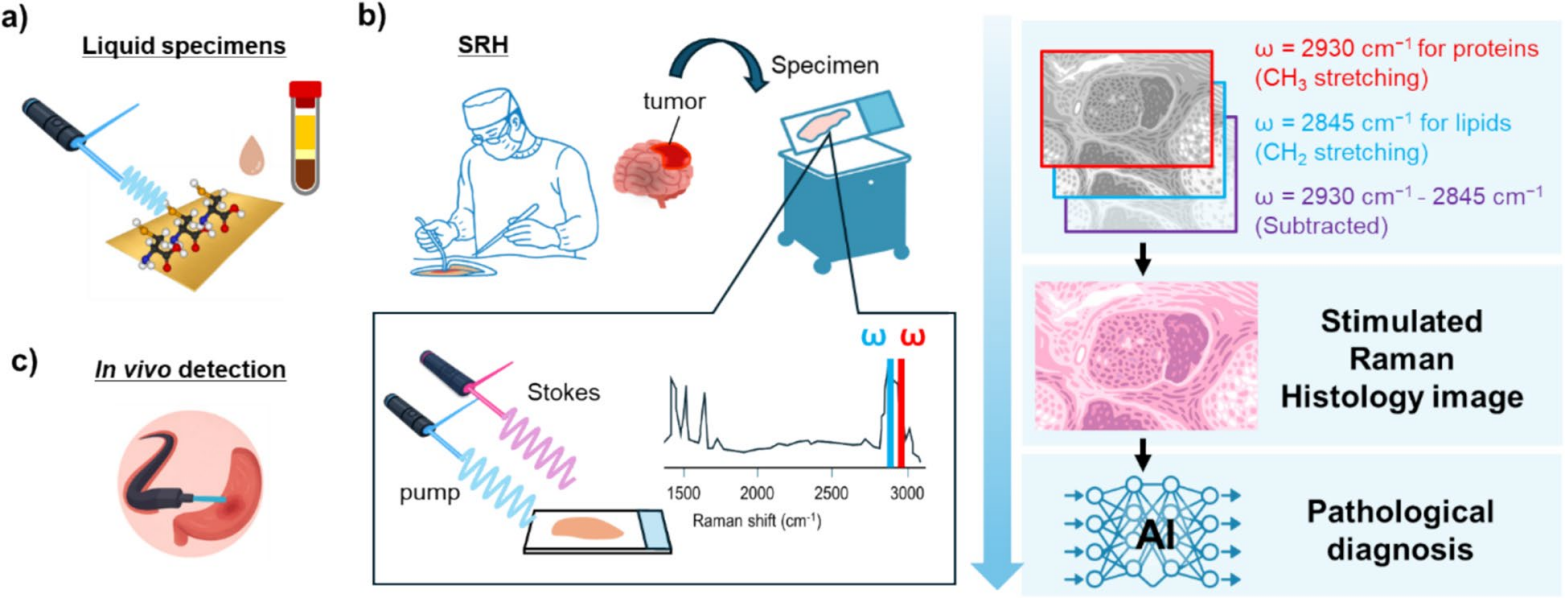
Medical application of Raman imaging. **a** SERS-based diagnosis using liquid samples such as serum and plasma. **b** Overview of stimulated Raman histology (SRH) workflow. Tumor samples collected from various tissues during surgery were placed on glass slides, and SRH images were acquired at two vibrational frequencies (ω_1_ = 2845 cm^−1^ for CH_2_ stretching for lipids, ω_2_ = 2930 cm^−1^ for CH_3_ stretching for proteins) to extract lipid/protein distributions of the samples with high compositional precision). The subtracted image (2930 cm^−1^–2845 cm^−1^) is also created, and from these, three images are post-processed to produce custom virtual H&E color images through pseudo-coloring. In AI-based molecular diagnostics, the entire slide image is divided into non-overlapping patches, each identifying areas exhibiting characteristic pathological findings and visualized as a heatmap. Pump and Stokes are two synchronized laser beams. When frequency difference matches to a molecular vibrational frequency, stimulated excitation of the vibrational transition occurs, resulting in strong SRS signal. **c **In vivo detection of disease by using fiber-optics Raman combined with endoscopy

When light interacts with a material, most of the scattered light has the same wavelength as the incident light (vibrational frequency, ω_0_, Rayleigh scattering), but a portion of the scattered light has a different wavelength (vibrational frequency, ω_0_—ω)—this wavelength shift is known as the Raman effect [39] (Fig. 1a). The Raman shift (ω) is a consequence of energy transfer to molecular rotational, vibrational, or electronic transitions, and thus, the Raman spectrum provides information about the species and states of the target molecules. Figure 1b shows an example of Raman spectrum of cells, which can be roughly divided into three regions: fingerprint region (< 1800 cm^−1^), silent region (1800–2800 cm^−1^) and CH stretching region (> 2800 cm^−1^). Raman peaks arise from molecular vibration of specific functional groups and functional groups present in biological samples mostly appear in the fingerprint region and the CH stretching region. On the other hand, signals arising from endogenous biological molecules are inherently absent in silent regions. In general, Raman scattering is very weak, necessitating lengthy observation times (e.g., several tens of minutes to several hours to acquire a single image). In order to increase the sensitivity, ingenious lighting techniques [40] were developed, and, more recently, measurements under cryo-conditions were conducted to minimize sample damage [41]. Other technical improvements included the development of microscopy techniques based on nonlinear Raman effects, such as coherent anti-Stokes Raman scattering (CARS) [13] and stimulated Raman scattering (SRS) [7]. These techniques achieve high sensitivity through the utilization of dual lasers (pump light and Stokes light, see also the legend of Fig. 2b), enabling high-speed Raman imaging at time scales of several seconds to several minutes, which is adequate for biological imaging.

An alternative approach to improve sensitivity is to combine vibrational transitions with other highly sensitive physical readouts. In 2019, a novel technique that converts vibrational information into fluorescent signals using stimulated Raman-excited fluorescence (SREF) was reported [42, 43]. This technology is based on a double resonance mechanism wherein vibrational excitation is performed using SRS, accompanied by electronic excitation with a third beam. This approach enables high-sensitivity detection without background interference in Raman microscopy, affording single-molecule sensitivity [42, 44]. Stimulated Raman photothermal microscopy (SRP) is another approach, based on the detection of refractive index changes induced by local heating due to the relaxation of SRS-induced vibrationally excited states [45].

## Label-free imaging of living systems using Raman microscopy

Label-free optical imaging captures the intrinsic properties of specimens, such as absorption, molecular vibrations, alterations in refractive index, autofluorescence, birefringence, and scattering, and utilizes them to generate imaging contrast [1, 46]. These techniques are non-invasive, and therefore preserve the molecular structural form and physiological functions, except where these may be affected by laser-induced phototoxicity. Some label-free imaging techniques are relatively easy to implement and are therefore widely accessible to biologists and clinicians [47]. Since the vibrational modes of molecules are determined by their chemical structures, Raman spectra are highly specific, and have been referred to as “molecular fingerprints.” Especially, the Raman fingerprint region (less than 1800 cm^−1^) and CH stretching region (above 2800 cm^−1^) are important for performing label-free imaging, since it contains a characteristic spectral pattern originating from intrinsic biomolecules. For instance, the Raman peak at 1655 cm⁻^1^ can be attributed to the amide I vibration commonly found in proteins, while Raman peak at 2850 cm^−1^ can be attributed to CH_2_ vibrations primarily contained in lipids (Fig. 1b). By using these regions, Raman imaging can characterize molecular species without labeling. This enables the visualization of a wide range of biomolecules [48] such as nucleic acids [49], as well as the intracellular distribution of drugs [50], the mapping of intracellular temperature profiles [51, 52], the analysis of higher-order structures of polypeptides [53], and the visualization of cellular aging by tracking changes in the wavenumber of amide bonds in proteins (Fig. 1c) [54].

### Medical application of Raman imaging

Advances in Raman analysis/method have led to potential medical application, which can be divided into three categories: (a) SERS-based diagnosis of liquid specimens, (b) analysis of tissue sample including clinical specimens, and (c) in vivo observation and diagnosis of lesions (Fig. 2). (a) SERS-based analysis of liquid specimens is a technique in which metal particles are mixed with liquid specimens such as serum and plasma to distinguish small differences in chemical composition between healthy and diseased specimens (changes in the quality and quantity of proteins, nucleic acids, etc., are reflected as changes in Raman wavenumber). This allows, for example, the identification of thyroid nodules from serum samples [55], coronary heart disease from urine samples [56], Alzheimer’s disease from tears [57], Parkinson’s disease [58], and COVID-19 from saliva [59]. In recent years, there have been reports that it can also be used to predict cancer progression using AI based on large amounts of data [60]. Although not covered in detail in this review, in addition to label-free detection, a variety of SERS-based method using small Raman tags, antibodies, or aptamers immobilized on metal surfaces have been developed to enable highly sensitive detection of specific biomarkers [61, 62]. (b) Spontaneous Raman analysis has been used for spectral analysis of clinical specimens with myocardial infarction [63] and for analyzing inflammatory diseases in mouse model [64]. It has also been applied for monitoring the cellular state transitions of mouse stem cells undergoing reprogramming, toward applications in regenerative medicine [65]. In recent years, stimulated Raman histology (SRH) method has been developed for rapid diagnosis which employs stimulated Raman scattering (SRS) to generate histology images similar to conventional H&E staining. The high resolution and the absence of a pre-processing requirement are particularly advantageous in intraoperative histology. The combination of SRH with artificial intelligence (AI) promises to be a game-changer in terms of increased accuracy and relieving the burden on histopathologists [32, 66]. (c) In vivo observation and diagnosis have become possible with fiber optic Raman technology, which can obtain the Raman spectral information of lesion parts and normal parts. Applications include gastric cancer diagnosis [67], oral cavity [68], laryngeal cancer [69], cervix [70], and esophageal cancer [71].

## Small Raman tags for probing cells and tissues at the molecular level

**Fig. 3:**
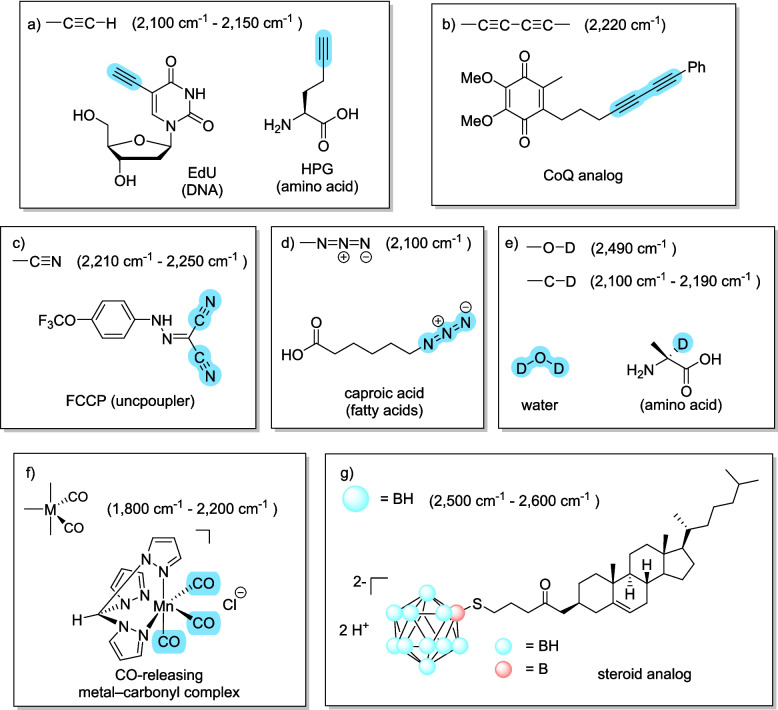
Representative vibrational probes for bioimaging. Small vibrational tags operating in the cell-silent region provide high specificity and optimal biocompatibility for bioimaging. Representative targets for metabolic imaging and drug imaging are shown, with the vibrational tags highlighted. **a** Alkyne tags [82]. **b** Diyne tags [82]. **c** Nitrile tags [83]. **d** Azide tags [82]. **e** Deuterium tags [81]. **f** Metal–carbonyl tags [84]. **g** B-H tags [85]

As discussed above, the Raman fingerprint region (less than 1800 cm^−1^) is important for performing label-free imaging, since it contains a characteristic spectral pattern of fundamental vibrational modes. However, peaks in this region often overlap in a complex manner, and thus, spectra must be carefully analyzed. In contrast, Raman imaging in the so-called silent region (1800–2800 cm^−1^), where signals arising from endogenous biological molecules are inherently absent, allows us to perform specific imaging with minimal background if small tags that generate signatures in the silent region have been introduced into target biomolecules (Fig. 1b) [72–75]. Since the first report of a small tag, namely 5-ethynyl-2'-deoxyuridine (EdU) [76], various small tags have enabled specific tracking of the target biomolecules with high specificity even within the living body (Fig. 3) [15, 18, 24, 77–79]. These tags should be as small as possible to minimize interference with biological functions, and so far deuterium (D) in C-D bonds [15, 80, 81], triple bonds such as alkynes or nitriles [82, 83], C≡O bonds [84], and B-H bonds [85], have been used as small tags to label target molecules.

### Probing intracellular phenomena

Since small tags minimize perturbations to biological systems [86], they have been used to investigate various intracellular phenomena. By generating signals proportional to the target bond concentration, small tags can visualize and quantify the spatial distribution and local abundance of bioactive molecules such as lipids [16, 18, 20, 30, 78, 87–95], nucleic acids [82, 96–99], amino acids [15, 16, 100, 101], peptides [102], proteins [15–17, 19, 101, 103, 104], sugars [22, 26, 27, 105–107], and cholesterol [23, 24, 78]. Compounds bearing a small Raman tag can be taken up by cells and tissues through multiple pathways, and some of them can be metabolically incorporated into biomolecules with high specificity [19, 21, 26, 81, 100, 108], enabling imaging with minimal biological impact [5, 109]. For example, spectral tracing of deuterium isotope (STRIDE) utilizes deuterated glucose, whose carbon–deuterium bonds are metabolically transferred to diverse macromolecules, such as DNA, protein, lipids, and glycogen. Thus, STRIDE can visualize metabolic dynamics and map newly synthesized macromolecules [26]. Deuterium oxide probing with stimulated Raman scattering (DO-SRS) [81] utilizes deuterated water (D_2_O), which can be metabolically incorporated into carbon–deuterium bonds of newly synthesized biomolecules such as lipids, proteins, carbohydrates, and nucleic acids. DO-SRS has been used as a probe for metabolic activities in a broad range of model organisms such as mice, *C. elegans*, microorganisms [81, 99, 110–113], and *Drosophila melanogaster* [112, 114, 115]. Besides deuterated analogues, triple bonds are useful due to their massive Raman cross-section and suitability for multiplexing [16, 82]. Furthermore, these small tags can be utilized for investigating diverse phenomena within cells. For example, by using the difference in Raman shifts arising from non-covalent interactions (e.g., electrostatic bonds and hydrogen bonds) in various solvents [116], phase transitions related to lipid toxicity in the endoplasmic reticulum membrane were demonstrated [30, 95]. Organelle-targeted Raman probes have been used for the rapid assessment of drug efficacy [117].

Raman tags introduced into drugs also have applications in pharmaceutical science [118]. For example, among drug candidates, ponatinib [119] and terbinafine [16] have an embedded alkyne structure, which can be utilized to evaluate intracellular distribution. Boron cluster compounds used for BNCT (boron neutron capture therapy) exhibit unique vibrational modes in the cell silent region, enabling direct imaging of cellular uptake of the boron cluster [120]. If such an intrinsic tag is not present, a small tag can be incorporated. For example, Mito-Q-*d*_15_, deuterated Mito-Q (a drug candidate) was used to evaluate mitochondrial uptake [121], and olaparib derivatives [122] with an introduced diyne structure have been used to investigate the intracellular distribution in cancer cells. In in vivo studies, alkyne-linked diynes are hallmark tags offering high sensitivity. For example, the primary binding sites of anisomycin [123], ferrostatin [124], antimycin-type depsipeptide [125], and JQ1 [126] have been studied using diyne-labeled analogues. SERS, which enhances weak Raman signals, is also potentially powerful for quantitative pharmacokinetic tracking [127].

### Applications to tissues and live organisms

Most alkynes or deuterium-based Raman probes can facilitate imaging at the tissue level as well as the cellular level [21], and important insights have been obtained into mouse tissue metabolism [21, 22] and disease states and drug responses in human organoids [128]. Furthermore, the application of Raman probes to model organisms such as nematodes and microorganisms [129] has clarified the significance of choline in nematode embryogenesis [18], as well as genetic factors controlling lipid distribution [89], molecular relationships between microbial metabolites and host metabolism [130], proteins newly synthesized during zebrafish embryogenesis in vivo [21], and the metabolism of deuterated or alkyne-derived fatty acids [131]. In addition, nanoparticle-based drug carriers incorporating Raman tags have enabled imaging with high specificity and sensitivity at the cellular [132] and tissue [133–135] levels. Further applications have been reported for photothermal therapy through the use of photothermal effects generated by laser irradiation [136].

## Strategies for high-sensitivity Raman imaging

**Fig. 4:**
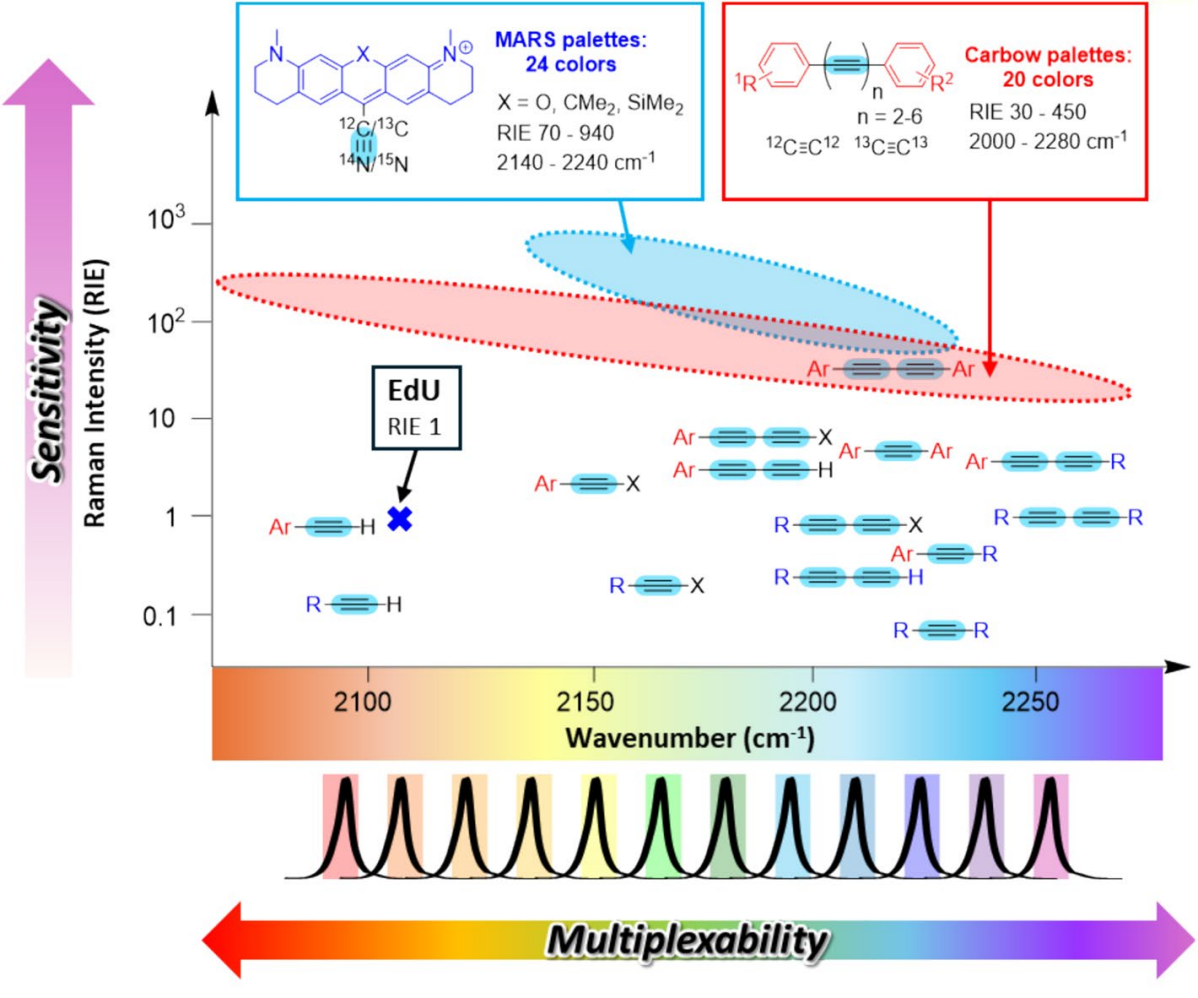
Structure–Raman shift/intensity relationship of alkynes. RIE values and Raman shift of alkynes can vary depending on the substituents (R, alkyl, alkenyl, acyl, etc.; X, halogen (Br or I), SiMe_3_; Ar, aryl) and the number of conjugated alkynes. Carbow dyes possess multiple alkynes with ultra-multiplexing capabilities [162]. MARS dyes exhibit extremely high RIE values due to the electronic pre resonance (epr) condition (epr-SRS), enabling highly sensitive detection [167]. Adapted from the reference [82]

Efforts to improve the sensitivity of Raman spectroscopy broadly fall into two categories: improved microscopy techniques (CARS, SRS, SERS) and molecular design. This section focuses on molecular design. Strategies include leveraging resonance effects (electronic interactions) and increasing the numbers of molecules, for example, by encapsulating them into beads (such as Rdot). In 2012, EdU was firstly used as a reference of Raman intensity for the calculation of relative Raman intensity versus EdU (RIE) (Figs. 3a, 4) [82]. Since this report, many researchers have used this benchmark for probe intensity.

Firstly, substitution is a valuable approach for enhancing Raman signals. For example, it was shown that modifying alkynes with substituents such as halogen, phenyl, carbonyl, and alkyne can enhance Raman intensity [70]. Currently, alkyne-linked diynes are widely used, and polyyne-based carbon rainbow dyes (Carbow dye) [137] which possesses multiple alkynes are a representative example utilizing this logic (Fig. 4, framed in red line). Besides such empirical approaches, quantum–mechanical calculations predicting Raman signal intensities [138–141] are helpful in the molecular design of Raman probes utilizing σ-π electronic interactions [140], n-π electronic interactions [139], and π-π electronic interactions [142].

In vibrational modes with high electron density [82, 137], electronic resonance effects can enhance the Raman signal by up to 10^6^-fold when the pump photon energy approaches the energy of the electronic state [143]. Furthermore, SRS imaging under the electronic pre resonance (epr) condition (epr-SRS) enables highly sensitive detection using near-infrared dyes such as Manhattan Raman scattering dyes (MARS dyes) [144] (Fig. 4, framed in blue line). Taking advantage of this high sensitivity together with the vibrational solvatochromism of these dyes, water heterogeneity within cells has been studied [145, 146]. Furthermore, in the case of spontaneous Raman imaging, fluorescence generally hinders Raman observation by overwhelming the Raman signals, and therefore, non-fluorescent resonance Raman probes such as those based on azobenzene units have been developed [147–149] to improve the sensitivity of spontaneous Raman microscopy. Recently, our group also reported cyano-hydrol green derivatives, which show suppressed fluorescence, and thus can be used for spontaneous Raman detection [150].

The combination of resonance enhancement with surface-enhanced Raman scattering (SERS) further boosts detection sensitivity to the single-molecule level [151–154]. SERS and tip-enhanced Raman scattering can amplify Raman signals by 10^6^ to 10^9^ times through localized plasmon excitation on metal surfaces. Utilizing click reactions to form nano assemblies is also effective [155]. However, there are toxicity concerns for biological samples with metal-based SERS substrates [156–159]. In order to enhance Raman scattering only with small molecules, the stacking-induced charge transfer Raman scattering (SICTERS) mechanism has been recently devised [160, 161], where small molecules self-assemble via π-conjugated stacking interactions, enabling three-dimensional charge transfer across adjacent molecules. The Raman scattering cross-sections are larger than those of SERS-based gold nanoprobes of similar size. This method can accelerate intraoperative detection of microtumors and non-invasive imaging of blood and lymph vessels. Furthermore, nanoparticles can be applied to locally enrich probe concentrations. Examples of this approach include covalently bonding Raman reporters to polymer backbones [162, 163] and techniques for concentrating dyes by doping commercial polystyrene nanoparticles [164–166]. Ultra-illuminated Raman-active nanoparticles (Rdots) containing a high density of reporter molecules also enable high-sensitivity detection for imaging cells and certain proteins [164].

## Raman probes for ultra-multiplexed observation

Although fluorescent probes possess a broad peak width, limiting the number of distinguishable colors to 2 to 5, this limitation can be overcome by employing cyclic immunofluorescence and mass cytometry [168], which enable multiplex imaging of up to dozens of biological targets. However, these methods are only applicable to fixed cells or thin tissue sections. In contrast, Raman spectroscopy not only provides high-resolution sharp signals that can be extensively multiplexed, but also is directly applicable to living cells [169, 170]. For example, 10-color multiplexed imaging using SERS gold nanoparticles was reported [171]. Imaging techniques were combined with advanced spectral analysis algorithms to obtain a hyper-multiplexed vibrational palette of over 20 colors and applied to profile cell-surface markers in the fingerprint region [165]. A recent study utilized highly sensitive SERS probes to distinguish 32 colors in the silent region [172] through one-shot 10-color SERS imaging of immune checkpoint proteins (ICPs) combined with correlation network analysis, revealing different expression patterns in different breast cancer subtypes [172]. Multiple ICPs, including CD47, LAG-3, and PD-L1, were identified as promising predictors for combination immunotherapy.

Isotope labeling is a potent strategy for generating more vibrational colors without affecting chemical properties. For example, the Raman frequency of terminal alkynes can be shifted from ~ 2120 to 2077 cm^−1^ and 2053 cm^−1^ through simple single or double ^13^C editing [29]. It has also been reported that the vibrational frequency of various Raman tags such as nitriles [42, 144, 150, 167], polyynes and polydiacetylenes [137, 173], cumulenes [174], and carbon nanotubes [175] can be tuned by isotope labeling. Through tag design and hyperspectral component deconvolution, multiple bioactive molecules can be analyzed [26, 81, 166, 176, 177]. MARS dyes, firstly reported in 2017, can be adjusted to afford over 20 vibrational colors within the cell-silent region, by isotope labeling, modification of core structures, ring expansion, and substitution effects, being one of the representative super-multiplexed Raman probes (Fig. 4) [144, 167]. MARS-based 8-color imaging of neural cell cultures revealed differences in DNA synthesis and protein metabolism between neurons and glial cells [144]. Furthermore, by combining MARS dyes and optimized tissue clearing methods, the Raman Dye Imaging and Tissue Clearing (RADIANT) method was developed to achieve one-shot multi-target imaging in millimeter-thick mice brain slices, revealing region-specific correlative networks and topologies during cerebral development [178].

Modifying the alkyne substituent patterns [82] and capping aryl groups [174] is another strategy to tune the wavenumber of the alkyne. Carbow dye, reported in 2018 (Fig. 4) [137], contains linearly conjugated alkynes, and by modifying the chain length, isotope labeling, and introducing terminal capping substituents, 20 Carbow dyes with distinct Raman peaks were produced in the cell-silent region, which enabled 10-color imaging of cellular structure [137], the highest multiplexing so far achieved in living cells. Compared to MARS, Carbow does not emit fluorescence in the visible region, and thus, it is suitable for multiplexed imaging in combination with fluorescence imaging. Specifically, an 8-color time-lapse imaging in live cells was achieved by integrating SRS and fluorescence imaging with a high-speed tuning system [179]. Furthermore, approximately 60,000 optical barcodes can be generated theoretically by encapsulating Carbow dyes in polymer beads [137]. Other than Carbow dyes, bespoke polymer nanoparticles have been engineered for multiplex imaging [163, 180]; these can be utilized for multiplex bioimaging by adjusting the amount of monomer added during copolymerization [181]. Besides such empirical approaches, quantum mechanical calculations predicting wavenumbers [123, 182] have also been used for the design of multiplexed Raman probes. As shown above, sensitivity and multiplexing capability of Raman probes are closely related, and have been evolved together.

## Functional Raman probes expand the scope of imaging

**Fig. 5:**
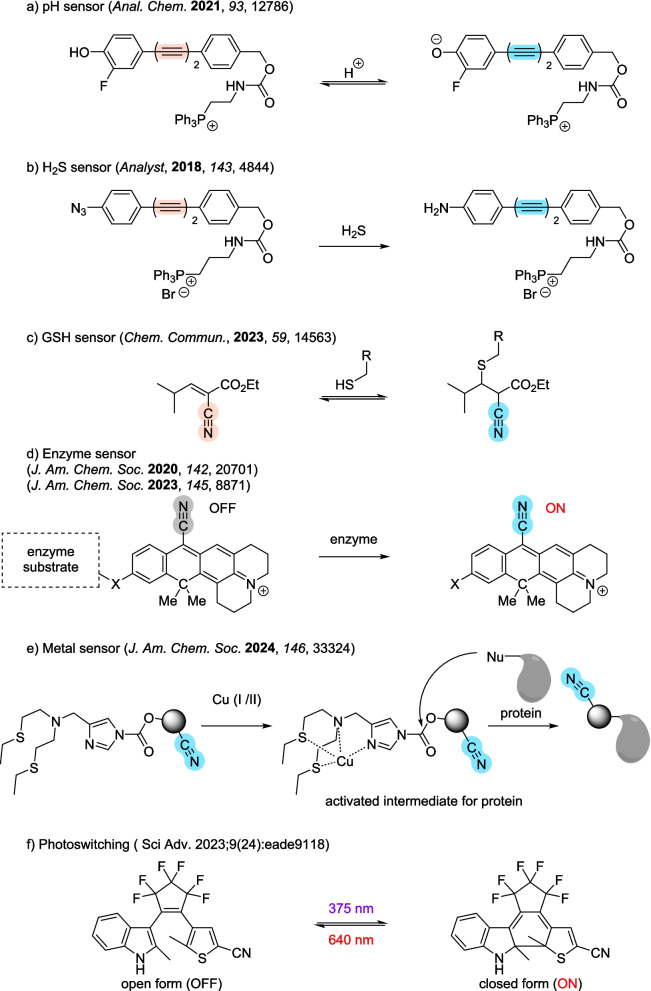
Mechanisms of representative Raman sensor probes. **a** pH-sensing Raman probes. **b** H_2_S-sensing Raman probes. **c** GSH-sensing Raman probes. **d** Enzyme-sensing Raman probes. **e** Metal-sensing Raman probes. **f** Photoswitching Raman probes

Most of the existing Raman probes exhibit constant Raman shift values and signal intensities, while in recent years, functional probes that respond to external stimuli have been developed for investigating biological phenomena in live cells, since activatable or switching logic can be fabricated by appropriate molecular design. Trifluoromethoxy carbonylcyanide phenylhydrazone (FCCP) was one of the first examples of a functional Raman probe; it shows a red shift of the nitrile stretching vibration upon deprotonation [83]. Since then, various probes responsive to different stimuli have been developed for monitoring biological phenomena in living cells (Fig. 5) [183], although the number of such probes is still limited compared to that of fluorescence probes. There are two main types of functional Raman probes. One is the ratio type that exhibits a shift in the Raman peak. The other is the activatable type that exhibits Raman signal enhancement. Other sensing strategies are also feasible, such as activity-based labeling [184], SERS [185–187], and enhancement via hotspot or nanocluster formation [155]. Some examples are presented below.

### pH sensing [188–191]

Ratiometric Raman sensors for various analytes have been developed for quantitative live-cell imaging based on the wavenumber shifts of butadiyne (Fig. 5a) [188, 189]. In particular, pH can be decoded from the frequency response of butadiyne bonded to functional groups with specific pKa values [188]. A similar approach can be applied to monitor enzyme activity in living cells [190]. pH sensors featuring nanoparticles have also been reported [191].

### Hydrogen sulfide sensing [192]

Hydrogen sulfide (H₂S) is a signaling molecule that is involved in numerous physiological processes [193, 194]. H_2_S is primarily generated in the cytoplasm and mitochondria from cysteine and homocysteine by enzymes such as cystathionine-γ-lyase (CSE) [195]. Physiological concentrations range from 0.1 to 300 μM, and abnormal levels are associated with various diseases, including Alzheimer’s disease [196]. Therefore, there is considerable interest in detecting H_2_S within living cells. A hydrogen sulfide sensor based on a bis-aryldiyne scaffold has been developed for this purpose. Reaction with hydrogen sulfide shifts the Raman peak of the diyne stretching mode, and changing the azide substituent to an amine group enables quantitative ratiometric measurements [192] (Fig. 5b).

### GSH sensing [197]

L-γ-glutamyl-L-cysteinylglycine (GSH) is a non-protein thiol synthesized in the cytoplasm as a defense against oxidative stress and nitrosative stress triggered by accumulation of free radicals. It protects the function of proteins vulnerable to oxidative damage and rescues cells from stress-induced apoptosis [198]. Intracellular GSH was successfully quantified by utilizing a reversible thia-Michael reaction using an α-cyanoacrylate (αCNA) derivative [197] (Fig. 5c).

### Enzyme activity sensing [174, 190, 199–201]

Our group has focused on the phenomenon that the pre-resonance Raman effect, exploited in MARS dyes, induces an exponential increase of Raman scattering intensity as the molecular absorption wavelength approaches the excitation wavelength of the microscope, and we developed a functional Raman probe capable of switching the Raman signal from off to on under epr-SRS microscopy (Fig. 5d). Furthermore, by incorporating isotope substitution on the CN group of 9CN-JCP, we discovered that the enzyme activity patterns differ in different types of cancer cell [199]. Targeting multiple enzyme activities may have clinical potential for the identification of cancer types. We expanded the molecular design based on rhodol derivatives, which have higher aggregation tendency compared to pyronine derivatives, and developed activatable Raman probes for enzymes with improved intracellular retention [200]. We realized that aggregation can enhance the Raman signal by increasing local dye concentration and recently developed functional Raman probes for enzyme activities that solely rely on aggregation-induced Raman signal generation [201]. Besides these probes, ratio type sensors have also been reported [174, 190], which show a shift in wavenumber upon reaction with target enzymes.

### Metal ion sensing [184]

Metal ions such as copper have roles in many biological functions [202], and Raman imaging can be employed to detect them (Fig. 5e) [184, 203]. Indeed, compared to conventional fluorescence imaging, vibrational probe-based monitoring is advantageous, since heavy metal ions may quench fluorescence signals but have minimal impact on vibrational probes. Furthermore, the inherent difficulty in detecting low concentrations of metal ions in intracellular metal ion pools can be circumvented by using Raman-specific sensitivity enhancement techniques (SERS or epr-SRS). Simultaneous imaging of redox species in cancer and neural cells can provide valuable insights into pathological mechanisms in lung cancer and neural cell death during cuproptosis.

### Photoswitching [204–209]

Vibrational probes can function as photo-responsive agents, exploiting their low perturbation, high multiplexing, and super-resolution imaging capabilities. For example, after the first report of a photoactivatable Raman tag based on cyclopropenone caging [204], we reported a photoactive Raman probe based on a pyronine skeleton [207]. Other photoswitchable vibrational probes, such as diarylethenes (DAEs), have also been reported [205, 206, 208]. Reversible and spatially selective multiplexed SRS imaging of organelles has been demonstrated with Carbow-based switches by pairing asymmetric DAE structures with a Carbow palette [206]. Moreover, super-resolution Raman imaging has been accomplished using a DAE-based photoswitchable Raman probe (Fig. 5f) [209]. Such vibrational optical switches with multiplexing capabilities represent new tools for investigating complex interactions and dynamics within biological systems.

## Conclusion and future prospects

In this review, we have covered the principles of Raman microscopy and provided examples of the latest developments in cutting-edge applications such as label-free imaging, small tags with minimal interference in living organisms, highly multiplexed Raman imaging, and methodology for achieving high-sensitivity imaging and molecular analysis. These developments in Raman microscopy have extended the toolset available for studying biological systems across multiple scales. Nevertheless, Raman microscopy still faces challenges such as insufficient sensitivity and slow imaging speed, along with limited availability, particularly for techniques based on nonlinear optical phenomena like SRS and CARS, due to the need for complex and expensive instrumentation. In addition, the variety of available Raman probes still remains limited compared to that of fluorescent probes, which restricts the range of applications in diverse biological contexts. Overcoming these limitations could expand the versatility of Raman microscopy and accelerate its translation into clinical practice, for example, for phenotypic screening and disease diagnosis. Future developments are expected to include AI-based image analysis of data from multiple imaging technologies, including Raman microscopy, within live cells to decipher complex intracellular relationships.

## Data Availability

Not applicable.

## Abbreviations

BNCT: Boron neutron capture therapy
CARS: Coherent anti-Stokes Raman scattering
EdU: 5-Ethynyl-2′-deoxyuridine
Epr: Electronic pre-resonance
RIE: Relative Raman intensity versus EdU
SERS: Surface-enhanced Raman scattering
SRH: Stimulated Raman histology
SRS: Stimulated Raman scattering

## References

[1] Shaked NT, Boppart SA, Wang LV, Popp J. Label-free biomedical optical imaging. Nat Photon. 2023;17(12):1031–41. 10.1038/s41566-023-01299-6 PMC10956740 38523771

[2] Edwards HGM, Vandenabeele P, Colomban P. Historical overview of Raman spectroscopy. In: Raman Spectroscopy in Cultural Heritage Preservation. Cultural Heritage Science. Switzerland AG: Springer Cham; 2023;2:7–18. 10.1007/978-3-031-14379-3_2.

[3] Turrell G, Corset J. Raman microscopy. San Diego, CA: Academic Press; 1996. 10.1016/b978-0-12-189690-4.x5018-2.

[4] Long DA. The Raman Effect: A Unified Treatment of the Theory of Raman Scattering by Molecules. New Jersey, NJ: John Wiley & Sons; 2003. 10.1002/0470845767.

[5] Benson S, de Moliner F, Tipping W, Vendrell M. Miniaturized chemical tags for optical imaging. Angew Chem Int Ed Engl. 2022;61(34):e202204788. 35704518 10.1002/anie.202204788 PMC9542129

[6] Freudiger CW, Min W, Saar BG, et al. Label-free biomedical imaging with high sensitivity by stimulated Raman scattering microscopy. Science. 2008;322(5909):1857–61. 19095943 10.1126/science.1165758 PMC3576036

[7] Ozeki Y, Dake F, Kajiyama S, Fukui K, Itoh K. Analysis and experimental assessment of the sensitivity of stimulated Raman scattering microscopy. Opt Express. 2009;17(5):3651–8. 19259205 10.1364/oe.17.003651

[8] Nandakumar P, Kovalev A, Volkmer A. Vibrational imaging based on stimulated raman scattering microscopy. New J Phys. 2009;11(3):033026.

[9] Min W, Cheng JX, Ozeki Y. Theory, innovations and applications of stimulated raman scattering microscopy. Nat Photon. 2025;19(8):803–16.

[10] Manifold B, Fu D. Quantitative stimulated raman scattering microscopy: promises and pitfalls. Annu Rev Anal Chem (Palo Alto Calif). 2022;15(1):269–89. 35300525 10.1146/annurev-anchem-061020-015110 PMC10083020

[11] Hill AH, Fu D. Cellular imaging using stimulated Raman scattering microscopy. Anal Chem. 2019;91(15):9333–42. 31287649 10.1021/acs.analchem.9b02095

[12] Duncan MD, Reintjes J, Manuccia TJ. Scanning coherent anti-Stokes Raman microscope. Opt Lett. 1982;7(8):350–2. 19714017 10.1364/ol.7.000350

[13] Zumbusch A, Holtom GR, Xie XS. Three-dimensional vibrational imaging by coherent anti-stokes Raman scattering. Phys Rev Lett. 1999;82(20):4142–5.

[14] Cheng JX, Volkmer A, Xie XS. Theoretical and experimental characterization of coherent anti-Stokes Raman scattering microscopy. J Opt Soc Am B. 2002;19(6):1363.

[15] Wei L, Yu Y, Shen Y, Wang MC, Min W. Vibrational imaging of newly synthesized proteins in live cells by stimulated Raman scattering microscopy. Proc Natl Acad Sci U S A. 2013;110(28):11226–31. 23798434 10.1073/pnas.1303768110 PMC3710790

[16] Wei L, Hu F, Shen Y, et al. Live-cell imaging of alkyne-tagged small biomolecules by stimulated Raman scattering. Nat Methods. 2014;11(4):410–2. 24584195 10.1038/nmeth.2878 PMC4040164

[17] Hong S, Chen T, Zhu Y, Li A, Huang Y, Chen X. Live-cell stimulated Raman scattering imaging of alkyne-tagged biomolecules. Angew Chem Int Ed Engl. 2014;53(23):5827–31. 24753329 10.1002/anie.201400328

[18] Hu F, Wei L, Zheng C, Shen Y, Min W. Live-cell vibrational imaging of choline metabolites by stimulated Raman scattering coupled with isotope-based metabolic labeling. Analyst. 2014;139(10):2312–7. 24555181 10.1039/c3an02281a PMC4069604

[19] Shen Y, Xu F, Wei L, Hu F, Min W. Live-cell quantitative imaging of proteome degradation by stimulated raman scattering. Angew Chem Int Ed Engl. 2014;53(22):5596–9. 24737659 10.1002/anie.201310725 PMC4231775

[20] Fu D, Yu Y, Folick A, et al. In vivo metabolic fingerprinting of neutral lipids with hyperspectral stimulated Raman scattering microscopy. J Am Chem Soc. 2014;136(24):8820–8. 24869754 10.1021/ja504199s PMC4073829

[21] Wei L, Shen Y, Xu F, et al. Imaging complex protein metabolism in live organisms by stimulated Raman scattering microscopy with isotope labeling. ACS Chem Biol. 2015;10(3):901–8. 25560305 10.1021/cb500787b PMC4610303

[22] Hu F, Chen Z, Zhang L, Shen Y, Wei L, Min W. Vibrational imaging of glucose uptake activity in live cells and tissues by stimulated raman scattering. Angew Chem Int Ed Engl. 2015;54(34):9821–5. 26207979 10.1002/anie.201502543 PMC4644272

[23] Lee HJ, Zhang W, Zhang D, et al. Assessing cholesterol storage in live cells and *C. elegans* by stimulated Raman scattering imaging of phenyl-Diyne cholesterol. Sci Rep. 2015;5(1):7930. 25608867 10.1038/srep07930 PMC4302291

[24] Alfonso-García A, Pfisterer SG, Riezman H, Ikonen E, Potma EO. D38-cholesterol as a raman active probe for imaging intracellular cholesterol storage. J Biomed Opt. 2016;21(6):61003. 26719944 10.1117/1.JBO.21.6.061003 PMC4681884

[25] Hu F, Shi L, Min W. Biological imaging of chemical bonds by stimulated Raman scattering microscopy. Nat Methods. 2019;16(9):830–42. 31471618 10.1038/s41592-019-0538-0

[26] Zhang L, Shi L, Shen Y, et al. Spectral tracing of deuterium for imaging glucose metabolism. Nat Biomed Eng. 2019;3(5):402–13. 31036888 10.1038/s41551-019-0393-4 PMC6599680

[27] Long R, Zhang L, Shi L, et al. Two-color vibrational imaging of glucose metabolism using stimulated raman scattering. Chem Commun (Camb). 2018;54(2):152–5. 29218356 10.1039/c7cc08217g PMC5764084

[28] Hong S, Chen T, Zhu Y, Li A, Huang Y, Chen X. Live‐cell stimulated raman scattering imaging of alkyne‐tagged biomolecules. Angew Chem Weinheim Bergstr Ger. 2014;126(23):5937–41. 10.1002/anie.201400328 24753329

[29] Chen Z, Paley DW, Wei L, et al. Multicolor live-cell chemical imaging by isotopically edited alkyne vibrational palette. J Am Chem Soc. 2014;136(22):8027–33. 24849912 10.1021/ja502706q PMC4063185

[30] Shen Y, Zhao Z, Zhang L, et al. Metabolic activity induces membrane phase separation in endoplasmic reticulum. Proc Natl Acad Sci U S A. 2017;114(51):13394–9. 29196526 10.1073/pnas.1712555114 PMC5754785

[31] Schultz C, Wegner T, Heusel C, et al. Alkyne-tagged imidazolium-based membrane cholesterol analogs for Raman imaging applications. Chem Sci. 2024;15(35):14323–35. 39156930 10.1039/d4sc03155e PMC11325193

[32] Kondepudi A, Pekmezci M, Hou X, et al. Foundation models for fast, label-free detection of glioma infiltration. Nature. 2025;637(8045):439–45. 39537921 10.1038/s41586-024-08169-3 PMC11711092

[33] Traynor D, Behl I, O’Dea D, et al. Raman spectral cytopathology for cancer diagnostic applications. Nat Protoc. 2021;16(7):3716–35. 34117476 10.1038/s41596-021-00559-5

[34] Wang T, Shi L. Bond-selective imaging at the frontier of biomedicine. Chem Biomed Imaging. 2025;(cbmi.5c00100). 10.1021/cbmi.5c00100. 10.1021/cbmi.5c00100 PMC12728753 41450471

[35] Cutshaw G, Uthaman S, Hassan N, Kothadiya S, Wen X, Bardhan R. The emerging role of Raman spectroscopy as an omics approach for metabolic profiling and biomarker detection toward precision medicine. Chem Rev. 2023;123(13):8297–346. 37318957 10.1021/acs.chemrev.2c00897 PMC10626597

[36] Tipping WJ, Wilson LT, Tomkinson NCO, Faulds K, Graham D. Label-free screening of drug-induced liver injury using stimulated Raman scattering microscopy and spectral phasor analysis. Anal Chem. 2024;96(26):10639–47. 38889191 10.1021/acs.analchem.4c01285 PMC11223099

[37] Helal KM, Taylor JN, Cahyadi H, et al. Raman spectroscopic histology using machine learning for nonalcoholic fatty liver disease. FEBS Lett. 2019;593(18):2535–44. 31254349 10.1002/1873-3468.13520

[38] Germond A, Ichimura T, Horinouchi T, Fujita H, Furusawa C, Watanabe TM. Raman spectral signature reflects transcriptomic features of antibiotic resistance in *Escherichia coli*. Commun Biol. 2018;1(1):85. 30271966 10.1038/s42003-018-0093-8 PMC6123714

[39] Patel PA, Shah MP, Raghani NR, Shah DD, Dhameliya TM. Introduction of basic theory and principle of Raman scattering and spectroscopy. In: Applied raman spectroscopy. Amsterdam, Elsevier Science. 2025:1–11. 10.1016/b978-0-443-21834-7.00001-3.

[40] Mochizuki K, Kumamoto Y, Maeda S, et al. High-throughput line-illumination Raman microscopy with multislit detection. Biomed Opt Express. 2023;14(3):1015–26. 36950233 10.1364/BOE.480611 PMC10026569

[41] Mizushima K, Kumamoto Y, Tamura S, et al. Raman microscopy of cryofixed biological specimens for high-resolution and high-sensitivity chemical imaging. Sci Adv. 2024;10(50):eadn0110. 39661690 10.1126/sciadv.adn0110 PMC11633761

[42] Xiong H, Shi L, Wei L, et al. Stimulated Raman excited fluorescence spectroscopy and imaging. Nat Photonics. 2019;13(6):412–7. 32607124 10.1038/s41566-019-0396-4 PMC7326316

[43] Qian N, Xiong H, Wei L, Shi L, Min W. Merging vibrational spectroscopy with fluorescence microscopy: combining the best of two worlds. Annu Rev Phys Chem. 2025;76(1):279–301. 39899841 10.1146/annurev-physchem-082423-121033

[44] Xiong H, Qian N, Miao Y, Zhao Z, Min W. Stimulated Raman excited fluorescence spectroscopy of visible dyes. J Phys Chem Lett. 2019;10(13):3563–70. 31185166 10.1021/acs.jpclett.9b01289 PMC6657358

[45] Zhu Y, Ge X, Ni H, et al. Stimulated Raman photothermal microscopy toward ultrasensitive chemical imaging. Sci Adv. 2023;9(43):eadi2181. 37889965 10.1126/sciadv.adi2181 PMC10610916

[46] Kumamoto Y, Harada Y, Takamatsu T, Tanaka H. Label-free molecular imaging and analysis by Raman spectroscopy. Acta Histochem Cytochem. 2018;51(3):101–10. 30083018 10.1267/ahc.18019 PMC6066646

[47] Murakami Y, Nuriya M, Hu Z, et al. Label-free visualization of ciliary rootlets in mouse brain. Anal Chem. 2025;97(27):14160–7. 40592769 10.1021/acs.analchem.4c05851

[48] Fujita K, Smith NI. Label-free molecular imaging of living cells. Mol Cells. 2008;26(6):530–5. 19306507

[49] Zhang X, Roeffaers MBJ, Basu S, et al. Label-free live-cell imaging of nucleic acids using stimulated Raman scattering microscopy. ChemPhysChem. 2012;13(4):1054–9. 22368112 10.1002/cphc.201100890 PMC3516876

[50] Machida M, Sugimura T, Kajimoto S, et al. Label-free tracking of nanoprodrug cellular uptake and metabolism using Raman and autofluorescence imaging. J Phys Chem B. 2023;127(17):3851–60. 37094294 10.1021/acs.jpcb.3c01133

[51] Sugimura T, Kajimoto S, Nakabayashi T. Label-free imaging of intracellular temperature by using the O-H stretching Raman band of water. Angew Chem Int Ed Engl. 2020;59(20):7755–60. 32048796 10.1002/anie.201915846

[52] Takahashi H, Kajimoto S, Irikura O, Nakabayashi T. Label-free intracellular temperature measurement by integrating Raman imaging and deep learning. Chem Lett. 2025;54(3). 10.1093/chemle/upaf032.

[53] Chaubey SK, Kumar R, Lalaguna PL, et al. Ultrasensitive Raman detection of biomolecular conformation at the attomole scale using chiral nanophotonics. Small. 2024;20(45):e2404536. 39045909 10.1002/smll.202404536

[54] Ishibashi S, Inoko A, Oka Y, Leproux P, Kano H. Coherent Raman microscopy visualizes ongoing cellular senescence through amide I peak shifts originating from β sheets in disordered nucleolar proteins. Sci Rep. 2024;14(1):27584. 39528609 10.1038/s41598-024-78899-x PMC11555345

[55] Xia L, Lu J, Chen Z, Cui X, Chen S, Pei D. Identifying benign and malignant thyroid nodules based on blood serum surface-enhanced Raman spectroscopy. Nanomedicine. 2021;32(102328):102328. 33181274 10.1016/j.nano.2020.102328

[56] Yang H, Zhao C, Li R, et al. Noninvasive and prospective diagnosis of coronary heart disease with urine using surface-enhanced Raman spectroscopy. Analyst. 2018;143(10):2235–42. 29577154 10.1039/c7an02022h

[57] Cennamo G, Montorio D, Morra VB, et al. Surface-enhanced Raman spectroscopy of tears: toward a diagnostic tool for neurodegenerative disease identification. J Biomed Opt. 2020;25(8):1–12. 32767890 10.1117/1.JBO.25.8.087002 PMC7406892

[58] Carlomagno C, Bertazioli D, Gualerzi A, et al. Identification of the Raman salivary fingerprint of Parkinson’s disease through the spectroscopic- computational combinatory approach. Front Neurosci. 2021;15:704963. 34764849 10.3389/fnins.2021.704963 PMC8576466

[59] Karunakaran V, Joseph MM, Yadev I, et al. A non-invasive ultrasensitive diagnostic approach for COVID-19 infection using salivary label-free SERS fingerprinting and artificial intelligence. J Photochem Photobiol B. 2022;234(112545):112545. 36049288 10.1016/j.jphotobiol.2022.112545 PMC9389522

[60] Shin H, Oh S, Hong S, et al. Early-stage lung cancer diagnosis by deep learning-based spectroscopic analysis of circulating exosomes. ACS Nano. 2020;14(5):5435–44. 32286793 10.1021/acsnano.9b09119

[61] Khristoforova Y, Bratchenko L, Bratchenko I. Raman-based techniques in medical applications for diagnostic tasks: a review. Int J Mol Sci. 2023;24(21):15605. 37958586 10.3390/ijms242115605 PMC10647591

[62] Tahir MA, Dina NE, Cheng H, Valev VK, Zhang L. Surface-enhanced Raman spectroscopy for bioanalysis and diagnosis. Nanoscale. 2021;13(27):11593–634. 34231627 10.1039/d1nr00708d

[63] Yamamoto T, Minamikawa T, Harada Y, et al. Label-free evaluation of myocardial infarct in surgically excised ventricular myocardium by Raman spectroscopy. Sci Rep. 2018;8(1):14671. 30279495 10.1038/s41598-018-33025-6 PMC6168494

[64] Lee SH, Lee SH, Shin JH, Choi S. Label‐free monitoring of inflammatory tissue conditions using a carrageenan‐induced acute inflammation rat model. Microsc Res Tech. 2018;81(6):544–50. 29473284 10.1002/jemt.23010

[65] Germond A, Panina Y, Shiga M, Niioka H, Watanabe TM. Following embryonic stem cells, their differentiated progeny, and cell-state changes during *iPS* reprogramming by Raman spectroscopy. Anal Chem. 2020;92(22):14915–23. 33112148 10.1021/acs.analchem.0c01800

[66] Hollon T, Jiang C, Chowdury A, et al. Artificial-intelligence-based molecular classification of diffuse gliomas using rapid, label-free optical imaging. Nat Med. 2023;29(4):828–32. 36959422 10.1038/s41591-023-02252-4 PMC10445531

[67] Duraipandian S, Sylvest Bergholt M, Zheng W, et al. Real-time Raman spectroscopy for in vivo, online gastric cancer diagnosis during clinical endoscopic examination. J Biomed Opt. 2012;17(8):081418. 23224179 10.1117/1.JBO.17.8.081418

[68] Bergholt MS, Zheng W, Huang Z. Characterizing variability in *in vivo* Raman spectroscopic properties of different anatomical sites of normal tissue in the oral cavity. J Raman Spectrosc. 2012;43(2):255–62.

[69] Lin K, Cheng DLP, Huang Z. Optical diagnosis of laryngeal cancer using high wavenumber Raman spectroscopy. Biosens Bioelectron. 2012;35(1):213–7. 22465448 10.1016/j.bios.2012.02.050

[70] Duraipandian S, Zheng W, Ng J, Low JJH, Ilancheran A, Huang Z. Simultaneous fingerprint and high-wavenumber confocal Raman spectroscopy enhances early detection of cervical precancer in vivo. Anal Chem. 2012;84(14):5913–9. 22724621 10.1021/ac300394f

[71] Bergholt MS, Zheng W, Lin K, et al. In vivo diagnosis of esophageal cancer using image-guided Raman endoscopy and biomolecular modeling. Technol Cancer Res Treat. 2011;10(2):103–12. 21381788 10.7785/tcrt.2012.500185

[72] Wang R, Wang Q, Zhou L. Triple-bond Raman probes: expanding molecular imaging in the cell-silent region. Next Nanotechnol. 2023;3–4(100022):100022.

[73] Vardaki MZ, Gregoriou VG, Chochos CL. Biomedical applications, perspectives and tag design concepts in the cell - silent Raman window. RSC Chem Biol. 2024;5(4):273–92. 38576725 10.1039/d3cb00217a PMC10989507

[74] Liu X, Liu X, Rong P, Liu D. Recent advances in background-free Raman scattering for bioanalysis. Trends Analyt Chem. 2020;123(115765):115765.

[75] Du J, Wang H, Wei L. Bringing vibrational imaging to chemical biology with molecular probes. ACS Chem Biol. 2022;17(7):1621–37. 35772040 10.1021/acschembio.2c00200 PMC10676805

[76] Yamakoshi H, Dodo K, Okada M, et al. Imaging of EdU, an alkyne-tagged cell proliferation probe, by Raman microscopy. J Am Chem Soc. 2011;133(16):6102–5. 21443184 10.1021/ja108404p

[77] van Manen HJ, Lenferink A, Otto C. Noninvasive imaging of protein metabolic labeling in single human cells using stable isotopes and Raman microscopy. Anal Chem. 2008;80(24):9576–82. 19006335 10.1021/ac801841y

[78] Matthäus C, Krafft C, Dietzek B, Brehm BR, Lorkowski S, Popp J. Noninvasive imaging of intracellular lipid metabolism in macrophages by Raman microscopy in combination with stable isotopic labeling. Anal Chem. 2012;84(20):8549–56. 22954250 10.1021/ac3012347

[79] Spratt SJ, Oguchi K, Miura K, et al. Probing methionine uptake in live cells by deuterium labeling and stimulated Raman scattering. J Phys Chem B. 2022;126(8):1633–9. 35195004 10.1021/acs.jpcb.1c08343

[80] Gaber BP, Yager P, Peticolas WL. Deuterated phospholipids as nonperturbing components for Raman studies of biomembranes. Biophys J. 1978;22(2):191–207. 580768 10.1016/S0006-3495(78)85484-8 PMC1473445

[81] Shi L, Zheng C, Shen Y, et al. Optical imaging of metabolic dynamics in animals. Nat Commun. 2018;9(1):2995. 30082908 10.1038/s41467-018-05401-3 PMC6079036

[82] Yamakoshi H, Dodo K, Palonpon A, et al. Alkyne-tag Raman imaging for visualization of mobile small molecules in live cells. J Am Chem Soc. 2012;134(51):20681–9. 23198907 10.1021/ja308529n

[83] Yamakoshi H, Palonpon AF, Dodo K, et al. Simultaneous imaging of protonated and deprotonated carbonylcyanide p-trifluoromethoxyphenylhydrazone in live cells by Raman microscopy. Chem Commun. 2014;50(11):1341–3. 10.1039/c3cc48587k 24346645

[84] Meister K, Niesel J, Schatzschneider U, Metzler-Nolte N, Schmidt DA, Havenith M. Label-free imaging of metal-carbonyl complexes in live cells by Raman microspectroscopy. Angew Chem Int Ed Engl. 2010;49(19):3310–2. 20349485 10.1002/anie.201000097

[85] Mochizuki M, Sato S, Asatyas S, Leśnikowski ZJ, Hayashi T, Nakamura H. Raman cell imaging with boron cluster molecules conjugated with biomolecules. RSC Adv. 2019;9(41):23973–8. 35530627 10.1039/c9ra04228h PMC9069464

[86] Dodo K, Tipping WJ, Yamakoshi H, et al. Alkyne-tag Raman imaging and sensing of bioactive compounds. Nat Rev Methods Primers. 2025;5(1):1–19.

[87] Stiebing C, Meyer T, Rimke I, et al. Real-time Raman and SRS imaging of living human macrophages reveals cell-to-cell heterogeneity and dynamics of lipid uptake. J Biophotonics. 2017;10(9):1217–26. 28164480 10.1002/jbio.201600279

[88] Weeks T, Schie I, den Hartigh LJ, Rutledge JC, Huser T. Lipid-cell interactions in human monocytes investigated by doubly-resonant coherent anti-Stokes Raman scattering microscopy. J Biomed Opt. 2011;16(2):021117. 21361680 10.1117/1.3544585 PMC3061331

[89] Yu Y, Mutlu AS, Liu H, Wang MC. High-throughput screens using photo-highlighting discover BMP signaling in mitochondrial lipid oxidation. Nat Commun. 2017;8(1):865. 29021566 10.1038/s41467-017-00944-3 PMC5636786

[90] Dodo K, Sato A, Tamura Y, et al. Synthesis of deuterated γ-linolenic acid and application for biological studies: metabolic tuning and Raman imaging. Chem Commun (Camb). 2021;57(17):2180–3. 33527102 10.1039/d0cc07824g

[91] Jia H, Liu J, Fang T, et al. The role of altered lipid composition and distribution in liver fibrosis revealed by multimodal nonlinear optical microscopy. Sci Adv. 2023;9(2):eabq2937. 36638165 10.1126/sciadv.abq2937 PMC9839333

[92] Li J, Condello S, Thomes-Pepin J, et al. Lipid desaturation is a metabolic marker and therapeutic target of ovarian cancer stem cells. Cell Stem Cell. 2017;20(3):303-314.e5. 28041894 10.1016/j.stem.2016.11.004 PMC5337165

[93] von Krusenstiern AN, Robson RN, Qian N, et al. Identification of essential sites of lipid peroxidation in ferroptosis. Nat Chem Biol. 2023;19(6):719–30. 36747055 10.1038/s41589-022-01249-3 PMC10238648

[94] Tan Y, Li J, Zhao G, et al. Metabolic reprogramming from glycolysis to fatty acid uptake and beta-oxidation in platinum-resistant cancer cells. Nat Commun. 2022;13(1):4554. 35931676 10.1038/s41467-022-32101-w PMC9356138

[95] Du J, Su Y, Qian C, et al. Raman-guided subcellular pharmaco-metabolomics for metastatic melanoma cells. Nat Commun. 2020;11(1):4830. 32973134 10.1038/s41467-020-18376-x PMC7518429

[96] Palonpon AF, Ando J, Yamakoshi H, et al. Raman and SERS microscopy for molecular imaging of live cells. Nat Protoc. 2013;8(4):677–92. 23471112 10.1038/nprot.2013.030

[97] Pliss A, Kuzmin AN, Kachynski AV, Prasad PN. Nonlinear optical imaging and Raman microspectrometry of the cell nucleus throughout the cell cycle. Biophys J. 2010;99(10):3483–91. 21081098 10.1016/j.bpj.2010.06.069 PMC2980749

[98] Watanabe H, Maehara D, Nishihara T, Tanabe K. Alkyne-tethered oligodeoxynucleotides that allow simultaneous detection of multiple DNA/RNA targets using Raman spectroscopy. RSC Adv. 2023;13(30):20756–60. 37441041 10.1039/d3ra03861k PMC10334030

[99] Bi X, Miao K, Wei L. Alkyne-tagged Raman probes for local environmental sensing by hydrogen-deuterium exchange. J Am Chem Soc. 2022;144(19):8504–14. 35508077 10.1021/jacs.2c01991

[100] Miao K, Wei L. Live-cell imaging and quantification of PolyQ aggregates by stimulated Raman scattering of selective deuterium labeling. ACS Cent Sci. 2020;6(4):478–86. 32341997 10.1021/acscentsci.9b01196 PMC7181319

[101] Zhang J, Yan S, He Z, et al. Small unnatural amino acid carried Raman tag for molecular imaging of genetically targeted proteins. J Phys Chem Lett. 2018;9(16):4679–85. 30067370 10.1021/acs.jpclett.8b01991

[102] Morgan DC, McDougall L, Knuhtsen A, et al. Raman active diyne-girder conformationally constrained p53 stapled peptides bind to MDM2 for visualisation without fluorophores. RSC Chem Biol. 2025;6(3):394–403. 39830683 10.1039/d4cb00288a PMC11741006

[103] Cai E, Chen Y, Zhang J, et al. Imaging specific proteins in living cells with small unnatural amino acid attached Raman reporters. Analyst. 2024;149(22):5476–81. 39400195 10.1039/d4an00758a

[104] Chen Y, Huang Z, Cai E, et al. Novel vibrational proteins. Anal Chem. 2024;96(42):16481–6. 39434664 10.1021/acs.analchem.4c01569

[105] de Moliner F, Knox K, Gordon D, et al. A palette of minimally tagged sucrose analogues for real-time Raman imaging of intracellular plant metabolism. Angew Chem Int Ed Engl. 2021;60(14):7637–42. 33491852 10.1002/anie.202016802 PMC8048481

[106] Karanja CW, Hong W, Younis W, Eldesouky HE, Seleem MN, Cheng JX. Stimulated Raman imaging reveals aberrant lipogenesis as a metabolic marker for azole-resistant *candida albicans*. Anal Chem. 2017;89(18):9822–9. 28813144 10.1021/acs.analchem.7b01798

[107] Lee D, Du J, Yu R, Su Y, Heath JR, Wei L. Visualizing subcellular enrichment of glycogen in live cancer cells by stimulated Raman scattering. Anal Chem. 2020;92(19):13182–91. 32907318 10.1021/acs.analchem.0c02348 PMC10676777

[108] Gazi E, Harvey TJ, Brown MD, Lockyer NP, Gardner P, Clarke NW. A FTIR microspectroscopic study of the uptake and metabolism of isotopically labelled fatty acids by metastatic prostate cancer. Vib Spectrosc. 2009;50(1):99–105.

[109] Azemtsop Matanfack G, Rüger J, Stiebing C, Schmitt M, Popp J. Imaging the invisible-bioorthogonal Raman probes for imaging of cells and tissues. J Biophotonics. 2020;13(9):e202000129. 32475014 10.1002/jbio.202000129

[110] Zhang M, Hong W, Abutaleb NS, et al. Rapid determination of antimicrobial susceptibility by stimulated Raman scattering imaging of D2O metabolic incorporation in a single bacterium. Adv Sci. 2020;7(19):2001452. 10.1002/advs.202001452 PMC7539191 33042757

[111] Ge X, Pereira FC, Mitteregger M, et al. SRS-FISH: a high-throughput platform linking microbiome metabolism to identity at the single-cell level. Proc Natl Acad Sci U S A. 2022;119(26):e2203519119. 35727976 10.1073/pnas.2203519119 PMC9245642

[112] Li Y, Zhang W, Fung AA, Shi L. DO-SRS imaging of diet regulated metabolic activities in Drosophila during aging processes. Aging Cell. 2022;21(4):e13586. 35257470 10.1111/acel.13586 PMC9009230

[113] Azemtsop Matanfack G, Taubert M, Guo S, et al. Monitoring deuterium uptake in single bacterial cells via two-dimensional Raman correlation spectroscopy. Anal Chem. 2021;93(21):7714–23. 34014079 10.1021/acs.analchem.1c01076

[114] Li Y, Zhang W, Fung AA, Shi L. DO-SRS imaging of metabolic dynamics in aging *Drosophila*. Analyst. 2021;146(24):7510–9. 34781326 10.1039/d1an01638e PMC10870347

[115] Li Y, Bagheri P, Chang P, et al. Direct imaging of lipid metabolic changes in Drosophila ovary during aging using DO-SRS microscopy. Front Aging. 2021;2:819903. 35822015 10.3389/fragi.2021.819903 PMC9261447

[116] Romei MG, von Krusenstiern EV, Ridings ST, et al. Frequency changes in terminal alkynes provide strong, sensitive, and solvatochromic Raman probes of biochemical environments. J Phys Chem B. 2023;127(1):85–94. 36538691 10.1021/acs.jpcb.2c06176 PMC9841980

[117] Adamczyk A, Tipping W, Mazuryk O, Graham D, Baranska M, Majzner K. Sensitive detection and identification method of erythrocyte-like cells upon doxorubicin induced differentiation with vibrational techniques. Anal Chem. 2025;97(31):16966–74. 40726176 10.1021/acs.analchem.5c02465 PMC12355469

[118] Dunnington EL, Wong BS, Fu D. Innovative approaches for drug discovery: quantifying drug distribution and response with Raman imaging. Anal Chem. 2024;96(20):7926–44. 38625100 10.1021/acs.analchem.4c01413 PMC11108735

[119] Sepp K, Lee M, Bluntzer MTJ, Helgason GV, Hulme AN, Brunton VG. Utilizing stimulated Raman scattering microscopy to study intracellular distribution of label-free ponatinib in live cells. J Med Chem. 2020;63(5):2028–34. 31829628 10.1021/acs.jmedchem.9b01546 PMC7073915

[120] Asai T, Liu H, Ozeki Y, Sato S, Hayashi T, Nakamura H. Imaging of cellular uptake of boron cluster compound by stimulated Raman scattering microscopy. Appl Phys Express. 2019;12(11):112004.

[121] Moriyama S, Mae M, Shibata D, et al. Multiple deuteration of triphenylphosphine and live-cell Raman imaging of deuterium-incorporated Mito-Q. Chem Commun (Camb). 2023;59(81):12100–3. 37721453 10.1039/d3cc04410f

[122] Steven CF, Punaha-Ravindra M, Lee M, et al. Application of stimulated raman scattering (SRS) microscopy for evaluation of olaparib biodistribution in an ovarian cancer cell line. Proc. SPIE. 2023;12357:1–10. 10.1117/12.2647240.

[123] Tipping WJ, Lee M, Serrels A, Brunton VG, Hulme AN. Imaging drug uptake by bioorthogonal stimulated Raman scattering microscopy. Chem Sci. 2017;8(8):5606–15. 30155229 10.1039/c7sc01837a PMC6103005

[124] Gaschler MM, Hu F, Feng H, Linkermann A, Min W, Stockwell BR. Determination of the subcellular localization and mechanism of action of ferrostatins in suppressing ferroptosis. ACS Chem Biol. 2018;13(4):1013–20. 29512999 10.1021/acschembio.8b00199 PMC5960802

[125] Seidel J, Miao Y, Porterfield W, et al. Structure-activity-distribution relationship study of anti-cancer antimycin-type depsipeptides. Chem Commun (Camb). 2019;55(63):9379–82. 31317975 10.1039/c9cc03051d PMC6675640

[126] Kawaguchi M, Yonetani Y, Mizuguchi T, et al. Visualization of modified bisarylbutadiyne-tagged small molecules in live-cell nuclei by stimulated Raman scattering microscopy. Anal Chem. 2024;96(17):6643–51. 38626411 10.1021/acs.analchem.3c05946

[127] Koike K, Bando K, Ando J, et al. Quantitative drug dynamics visualized by alkyne-tagged plasmonic-enhanced Raman microscopy. ACS Nano. 2020;14(11):15032–41. 33079538 10.1021/acsnano.0c05010

[128] Xu FX, Sun R, Owens R, Hu K, Fu D. Assessing drug uptake and response differences in 2D and 3D cellular environments using stimulated Raman scattering microscopy. Anal Chem. 2024;96(36):14480–9. 39186736 10.1021/acs.analchem.4c02592 PMC12358159

[129] Yasuda M, Takeshita N, Shigeto S. Deuterium-labeled Raman tracking of glucose accumulation and protein metabolic dynamics in *Aspergillus nidulans* hyphal tips. Sci Rep. 2021;11(1):1279. 33446770 10.1038/s41598-020-80270-9 PMC7809412

[130] Lin CCJ, Wang MC. Microbial metabolites regulate host lipid metabolism through NR5A-Hedgehog signalling. Nat Cell Biol. 2017;19(5):550–7. 28436966 10.1038/ncb3515 PMC5635834

[131] Law SSY, Asanuma M, Shou J, Ozeki Y, Kodama Y, Numata K. Deuterium- and alkyne-based bioorthogonal Raman probes for in situ quantitative metabolic imaging of lipids within plants. JACS Au. 2023;3(6):1604–14. 37388682 10.1021/jacsau.3c00041 PMC10302745

[132] Matthäus C, Kale A, Chernenko T, Torchilin V, Diem M. New ways of imaging uptake and intracellular fate of liposomal drug carrier systems inside individual cells, based on Raman microscopy. Mol Pharm. 2008;5(2):287–93. 18197626 10.1021/mp7001158 PMC2715828

[133] Wei M, Qian N, Gao X, Lang X, Song D, Min W. Single-particle imaging of nanomedicine entering the brain. Proc Natl Acad Sci U S A. 2024;121(5):e2309811121. 38252832 10.1073/pnas.2309811121 PMC10835139

[134] Vanden-Hehir S, Cairns SA, Lee M, et al. Alkyne-tagged PLGA allows direct visualization of nanoparticles in vitro and ex vivo by stimulated Raman scattering microscopy. Biomacromol. 2019;20(10):4008–14. 10.1021/acs.biomac.9b01092 PMC6794644 31408325

[135] Lang X, Gao X, Wei M, Qian N, Min W. Bioorthogonal chemical imaging of solid lipid nanoparticles with minimal labeling by stimulated Raman scattering microscopy. Nat Sci. 2023;3(2):e202103304.

[136] Wang J, Liang D, Jin Q, Feng J, Tang X. Bioorthogonal SERS nanotags as a precision theranostic platform for in vivo SERS imaging and cancer photothermal therapy. Bioconjug Chem. 2020;31(2):182–93. 31940174 10.1021/acs.bioconjchem.0c00022

[137] Hu F, Zeng C, Long R, et al. Supermultiplexed optical imaging and barcoding with engineered polyynes. Nat Methods. 2018;15(3):194–200. 29334378 10.1038/nmeth.4578 PMC5831481

[138] Neugebauer J, Reiher M, Kind C, Hess BA. Quantum chemical calculation of vibrational spectra of large molecules–Raman and IR spectra for Buckminsterfullerene. J Comput Chem. 2002;23(9):895–910. 11984851 10.1002/jcc.10089

[139] Li Y, Townsend KM, Dorn RS, Prescher JA, Potma EO. Enhancing alkyne-based Raman tags with a sulfur linker. J Phys Chem B. 2023;127(9):1976–82. 36821830 10.1021/acs.jpcb.2c09093

[140] Yamakita Y, Isogai Y, Ohno K. Large Raman-scattering activities for the low-frequency modes of substituted benzenes: induced polarizability and stereo-specific ring-substituent interactions. J Chem Phys. 2006;124(10):104301. 16542073 10.1063/1.2163344

[141] Torii H, Ishikawa A, Tasumi M. Electron-vibration interaction and the Raman intensities of a quinoid molecule. J Mol Struct. 1997;413–414:73–9.

[142] Tommasini M, Milani A, Fazzi D, et al. Π-conjugation and end group effects in long cumulenes: Raman spectroscopy and DFT calculations. J Phys Chem C Nanomater Interfaces. 2014;118(45):26415–25.

[143] Hirakawa AY, Tsuboi M. Molecular geometry in an excited electronic state and a preresonance Raman effect. Science. 1975;188(4186):359–61. 17807877 10.1126/science.188.4186.359

[144] Wei L, Chen Z, Shi L, et al. Super-multiplex vibrational imaging. Nature. 2017;544(7651):465–70. 28424513 10.1038/nature22051 PMC5939925

[145] Shi L, Hu F, Min W. Optical mapping of biological water in single live cells by stimulated Raman excited fluorescence microscopy. Nat Commun. 2019;10(1):4764. 31628307 10.1038/s41467-019-12708-2 PMC6802100

[146] Lang X, Welsher K. Mapping solvation heterogeneity in live cells by hyperspectral stimulated Raman scattering microscopy. J Chem Phys. 2020;152(17):174201. 32384848 10.1063/1.5141422

[147] Tang Y, Zhuang Y, Zhang S, et al. Azo-enhanced Raman scattering for enhancing the sensitivity and tuning the frequency of molecular vibrations. ACS Cent Sci. 2021;7(5):768–80. 34079895 10.1021/acscentsci.1c00117 PMC8161494

[148] Watanabe H, Maehara D, Nishihara T, Tanabe K. Raman signal enhancement by DABCYL-substitution on DNA aptamer for identification of cellular ATP. Bioconjug Chem. 2022;33(12):2314–9. 36468974 10.1021/acs.bioconjchem.2c00541

[149] Kuzmin AN, Pliss A, Lim CK, et al. Resonance Raman probes for organelle-specific labeling in live cells. Sci Rep. 2016;6:28483. 27339882 10.1038/srep28483 PMC4919686

[150] Fujioka H, Murao Y, Okinaka M, et al. Cyano-Hydrol green derivatives: expanding the 9-cyanopyronin-based resonance Raman vibrational palette. Bioorg Med Chem Lett. 2024;106(129757):129757. 38636718 10.1016/j.bmcl.2024.129757

[151] Nie S, Emory SR. Probing single molecules and single nanoparticles by surface-enhanced Raman scattering. Science. 1997;275(5303):1102–6. 9027306 10.1126/science.275.5303.1102

[152] Kneipp K, Wang Y, Kneipp H, et al. Single molecule detection using surface-enhanced Raman scattering (SERS). Phys Rev Lett. 1997;78(9):1667–70.

[153] Demirel G, Gieseking RLM, Ozdemir R, et al. Molecular engineering of organic semiconductors enables noble metal-comparable SERS enhancement and sensitivity. Nat Commun. 2019;10(1):5502. 31796731 10.1038/s41467-019-13505-7 PMC6890673

[154] Qiu C, Cheng Z, Lv C, Wang R, Yu F. Development of bioorthogonal SERS imaging probe in biological and biomedical applications. Chin Chem Lett. 2021;32(8):2369–79.

[155] Zhu W, Wang CY, Hu JM, Shen AG. Promoted “click” SERS detection for precise intracellular imaging of caspase-3. Anal Chem. 2021;93(11):4876–83. 33660989 10.1021/acs.analchem.0c04997

[156] Lane LA, Qian X, Nie S. SERS nanoparticles in medicine: from label-free detection to spectroscopic tagging. Chem Rev. 2015;115(19):10489–529. 26313254 10.1021/acs.chemrev.5b00265

[157] Liu Y, Zheng Y, Chen XH, et al. Fundamental theory of biodegradable metals—definition, criteria, and design. Adv Funct Mater. 2019;29(18):1805402.

[158] Fadeel B, Garcia-Bennett AE. Better safe than sorry: understanding the toxicological properties of inorganic nanoparticles manufactured for biomedical applications. Adv Drug Deliv Rev. 2010;62(3):362–74. 19900497 10.1016/j.addr.2009.11.008

[159] Maiti KK, Dinish US, Samanta A, et al. Multiplex targeted in vivo cancer detection using sensitive near-infrared SERS nanotags. Nano Today. 2012;7(2):85–93.

[160] Zhang Y, Zhang W, Qiu Y, et al. Molecular engineering of a SICTERS small molecule with superior in vivo Raman imaging and photothermal performance. J Am Chem Soc. 2025;147(12):10247–59. 40073295 10.1021/jacs.4c16411

[161] Gao S, Zhang Y, Cui K, et al. Self-stacked small molecules for ultrasensitive, substrate-free Raman imaging in vivo. Nat Biotechnol. 2025;43(6):936–47. 39169265 10.1038/s41587-024-02342-9 PMC12167709

[162] Tian S, Li H, Li Z, et al. Polydiacetylene-based ultrastrong bioorthogonal Raman probes for targeted live-cell Raman imaging. Nat Commun. 2020;11(1):81. 31900403 10.1038/s41467-019-13784-0 PMC6941979

[163] Jin Q, Fan X, Chen C, Huang L, Wang J, Tang X. Multicolor raman beads for multiplexed tumor cell and tissue imaging and in vivo tumor spectral detection. Anal Chem. 2019;91(6):3784–9. 30758186 10.1021/acs.analchem.9b00028

[164] Zhao Z, Chen C, Wei S, et al. Ultra-bright raman dots for multiplexed optical imaging. Nat Commun. 2021;12(1):1305. 33637723 10.1038/s41467-021-21570-0 PMC7910594

[165] Nishiyama R, Hiramatsu K, Kawamura S, et al. Color-scalable flow cytometry with Raman tags. PNAS Nexus. 2023;2(2):gad001. 10.1093/pnasnexus/pgad001 PMC9950787 36845353

[166] Chen C, Zhao Z, Qian N, Wei S, Hu F, Min W. Multiplexed live-cell profiling with raman probes. Nat Commun. 2021;12(1):3405. 34099708 10.1038/s41467-021-23700-0 PMC8184955

[167] Miao Y, Qian N, Shi L, Hu F, Min W. 9-cyanopyronin probe palette for super-multiplexed vibrational imaging. Nat Commun. 2021;12(1):4518. 34312393 10.1038/s41467-021-24855-6 PMC8313527

[168] Bodenmiller B. Multiplexed epitope-based tissue imaging for discovery and healthcare applications. Cell Syst. 2016;2(4):225–38. 27135535 10.1016/j.cels.2016.03.008

[169] Li Y, Sun Y, Shi L. Viewing 3D spatial biology with highly-multiplexed Raman imaging: from spectroscopy to biotechnology. Chem Commun (Camb). 2024;60(66):8658–69. 39041798 10.1039/d4cc02319f

[170] Miao Y, Shi L, Hu F, Min W. Probe design for super-multiplexed vibrational imaging. Phys Biol. 2019;16(4):041003. 30870829 10.1088/1478-3975/ab0fcd

[171] Zavaleta CL, Smith BR, Walton I, et al. Multiplexed imaging of surface enhanced raman scattering nanotags in living mice using noninvasive raman spectroscopy. Proc Natl Acad Sci U S A. 2009;106(32):13511–6. 19666578 10.1073/pnas.0813327106 PMC2726370

[172] Li J, Liu F, Bi X, Ye J. Imaging immune checkpoint networks in cancer tissues with supermultiplexed SERS nanoprobes. Biomaterials. 2023;302(122327):122327. 37716283 10.1016/j.biomaterials.2023.122327

[173] Li M, Tian S, Meng F, et al. Continuously multiplexed ultrastrong raman probes by precise isotopic polymer backbone doping for multidimensional information storage and encryption. Nano Lett. 2022;22(11):4544–51. 35604007 10.1021/acs.nanolett.2c01443

[174] Bai X, Zhang R, Yang Y, Hu F. Live-cell multiplexed imaging and chemical sensing with cumulene and polyyne allotropes. Anal Chem. 2025;97(28):15393–401. 40557718 10.1021/acs.analchem.5c02365

[175] Liu Z, Li X, Tabakman SM, Jiang K, Fan S, Dai H. Multiplexed multicolor raman imaging of live cells with isotopically modified single walled carbon nanotubes. J Am Chem Soc. 2008;130(41):13540–1. 18803379 10.1021/ja806242t PMC2617744

[176] Zhao Z, Chen C, Xiong H, Ji J, Min W. Metabolic activity phenotyping of single cells with multiplexed vibrational probes. Anal Chem. 2020;92(14):9603–12. 32530266 10.1021/acs.analchem.0c00790

[177] Liu X, Shi L, Zhao Z, Shu J, Min W. VIBRANT: spectral profiling for single-cell drug responses. Nat Methods. 2024;21(3):501–11. 38374266 10.1038/s41592-024-02185-x PMC11214684

[178] Shi L, Wei M, Miao Y, et al. Highly-multiplexed volumetric mapping with raman dye imaging and tissue clearing. Nat Biotechnol. 2022;40(3):364–73. 34608326 10.1038/s41587-021-01041-z PMC8930416

[179] Shou J, Oda R, Hu F, et al. Super-multiplex imaging of cellular dynamics and heterogeneity by integrated stimulated Raman and fluorescence microscopy. iScience. 2021;24(8):102832. 34381966 10.1016/j.isci.2021.102832 PMC8333161

[180] Hu F, Brucks SD, Lambert TH, Campos LM, Min W. Stimulated Raman scattering of polymer nanoparticles for multiplexed live-cell imaging. Chem Commun (Camb). 2017;53(46):6187–90. 28474031 10.1039/c7cc01860f PMC5623589

[181] Zhu W, Cai EL, Li HZ, et al. Precise encoding of triple-bond Raman scattering of single polymer nanoparticles for multiplexed imaging application. Angew Chem Int Ed Engl. 2021;60(40):21846–52. 34227191 10.1002/anie.202106136

[182] Streu K, Hunsberger S, Patel J, Wan X, Daly CA Jr. Development of a universal method for vibrational analysis of the terminal alkyne C≡C stretch. J Chem Phys. 2024;160(7). 10.1063/5.0185580. 10.1063/5.0185580 38364010

[183] Liao SJ, Cao J, Zhu W, Li W, Hu JM, Shen AG. Recent progress of responsive Raman scattering probes for biosensing and bioimaging. Trends Analyt Chem. 2023;169(117357):117357.

[184] Jiang Y, El Khoury E, Pezacki AT, et al. An activity-based sensing approach to multiplex mapping of labile copper pools by stimulated raman scattering. J Am Chem Soc. 2024;146(49):33324–37. 39586074 10.1021/jacs.4c06296 PMC11844218

[185] Tanwar S, Kim JH, Bulte JWM, Barman I. Surface-enhanced Raman scattering: an emerging tool for sensing cellular function. Wiley Interdiscip Rev Nanomed Nanobiotechnol. 2022;14(4):e1802. 35510405 10.1002/wnan.1802 PMC9302385

[186] Wang X, Wang J, Xu S. Microdroplet-SERS platform for single cell-secreted VEGF and extracellular pH analysis in oxidative stress event. Sens Actuators B Chem. 2025;422(136545):136545.

[187] Makanai H, Nishihara T, Tanabe K. Surface-enhanced raman scattering identification of nucleic acid targets by acetylene-tagged Hoechst molecule binding with DNA-tethered gold nanoparticles. ACS Appl Nano Mater. 2022;5(2):2935–42.

[188] Wilson LT, Tipping WJ, Jamieson LE, et al. A new class of ratiometric small molecule intracellular pH sensors for raman microscopy. Analyst. 2020;145(15):5289–98. 32672252 10.1039/d0an00865f

[189] Wilson LT, Tipping WJ, Wetherill C, et al. Mitokyne: a ratiometric raman probe for mitochondrial pH. Anal Chem. 2021;93(37):12786–92. 34505518 10.1021/acs.analchem.1c03075

[190] Braddick HJ, Tipping WJ, Wilson LT, et al. Determination of intracellular esterase activity using ratiometric raman sensing and spectral phasor analysis. Anal Chem. 2023;95(12):5369–76. 36926851 10.1021/acs.analchem.2c05708 PMC10061367

[191] Shen Y, Liang L, Zhang S, et al. Organelle-targeting surface-enhanced raman scattering (SERS) nanosensors for subcellular pH sensing. Nanoscale. 2018;10(4):1622–30. 29239454 10.1039/c7nr08636a

[192] Zeng C, Hu F, Long R, Min W. A ratiometric raman probe for live-cell imaging of hydrogen sulfide in mitochondria by stimulated raman scattering. Analyst. 2018;143(20):4844–8. 30246812 10.1039/c8an00910d PMC6249677

[193] Gadalla MM, Snyder SH. Hydrogen sulfide as a gasotransmitter. J Neurochem. 2010;113(1):14–26. 20067586 10.1111/j.1471-4159.2010.06580.x PMC2965526

[194] Kimura H. Hydrogen sulfide: its production and functions. Exp Physiol. 2011;96(9):833–5. 21527544 10.1113/expphysiol.2011.057455

[195] Wallace JL, Wang R. Hydrogen sulfide-based therapeutics: exploiting a unique but ubiquitous gasotransmitter. Nat Rev Drug Discov. 2015;14(5):329–45. 25849904 10.1038/nrd4433

[196] Eto K, Asada T, Arima K, Makifuchi T, Kimura H. Brain hydrogen sulfide is severely decreased in Alzheimer’s disease. Biochem Biophys Res Commun. 2002;293(5):1485–8. 12054683 10.1016/S0006-291X(02)00422-9

[197] Yamakoshi H, Shibata D, Bando K, et al. Ratiometric analysis of reversible thia-Michael reactions using nitrile-tagged molecules by Raman microscopy. Chem Commun (Camb). 2023;59(98):14563–6. 37986604 10.1039/d3cc05015g

[198] Raj Rai S, Bhattacharyya C, Sarkar A, et al. Glutathione: role in oxidative/nitrosative stress, antioxidant defense, and treatments. ChemistrySelect. 2021;6(18):4566–90.

[199] Fujioka H, Shou J, Kojima R, Urano Y, Ozeki Y, Kamiya M. Multicolor activatable raman probes for simultaneous detection of plural enzyme activities. J Am Chem Soc. 2020;142(49):20701–7. 33225696 10.1021/jacs.0c09200

[200] Fujioka H, Kawatani M, Spratt SJ, et al. Activatable raman probes utilizing enzyme-induced aggregate formation for selective ex vivo imaging. J Am Chem Soc. 2023;145(16):8871–81. 37057960 10.1021/jacs.2c12381 PMC10141441

[201] Okinaka M, Kawatani M, Fujioka H, et al. Functional raman probes for detecting enzyme activities based on aggregation control. Anal Chem. 2025;97(35):19057–65. 40859802 10.1021/acs.analchem.5c02231 PMC12424018

[202] Lippard SJ, Berg JM. Principles of bioinorganic chemistry. Mill Valley CA: University Science Books; 1994.

[203] Feng H, Fu Q, Du W, et al. Quantitative assessment of copper(II) in Wilson’s disease based on photoacoustic imaging and ratiometric surface-enhanced raman scattering. ACS Nano. 2021;15(2):3402–14. 33508938 10.1021/acsnano.0c10407

[204] Du J, Wei L. Multicolor photoactivatable raman probes for subcellular imaging and tracking by cyclopropenone caging. J Am Chem Soc. 2022;144(2):777–86. 34913693 10.1021/jacs.1c09689

[205] Ao J, Fang X, Miao X, et al. Switchable stimulated raman scattering microscopy with photochromic vibrational probes. Nat Commun. 2021;12(1):3089. 34035304 10.1038/s41467-021-23407-2 PMC8149663

[206] Yang Y, Bai X, Hu F. Photoswitchable polyynes for multiplexed stimulated raman scattering microscopy with reversible light control. Nat Commun. 2024;15(1):2578. 38519503 10.1038/s41467-024-46904-6 PMC10959996

[207] Kawatani M, Spratt SJ, Fujioka H, et al. 9-cyano-10-telluriumpyronin derivatives as red-light-activatable Raman probes. Chem Asian J. 2023;18(2):e202201086. 36461627 10.1002/asia.202201086 PMC10107100

[208] Shou J, Ozeki Y. Photoswitchable stimulated raman scattering spectroscopy and microscopy. Opt Lett. 2021;46(9):2176–9. 33929447 10.1364/OL.418240

[209] Shou J, Komazawa A, Wachi Y, et al. Super-resolution vibrational imaging based on photoswitchable Raman probe. Sci Adv. 2023;9(24):eade9118. 37327330 10.1126/sciadv.ade9118 PMC10275589

